# Immunoinformatics-driven design of multi-epitope vaccine targeting antibiotic-resistant *Salmonella typhimurium*

**DOI:** 10.1371/journal.pone.0342426

**Published:** 2026-02-26

**Authors:** Adnan Khan, Ayaz Ahmad, Farhad Badshah, Muhammad Ali, Muhammad Salman Khan, Warda Naz, Eliana Ibánez-Arancibia, Patricio R. De los Ríos-Escalante, Mohamed Taha Yassin

**Affiliations:** 1 Department of Biotechnology, Abdul Wali Khan University Mardan, Mardan, Khyber Pakhtunkhwa, Pakistan; 2 Livestock and Poultry Multi-Omics of MARA, Agricultural Genomics Institute at Shenzhen, Chinese Academy of Agricultural Sciences, Shenzhen, China; 3 State Key Laboratory of Animal Biotech Breeding, Institute of Animal Sciences, Chinese Academy of Agricultural Sciences, Beijing, China; 4 Department of Zoology, Abdul Wali Khan University Mardan, Mardan, Pakistan; 5 Department of Zoology, Hazara University Mansehra, Dhodial, Khyber Pakhtunkhwa, Pakistan; 6 PhD Program in Sciences Mentioning Applied Molecular and Cell Biology, La Frontera University, Temuco, Chile; 7 Laboratory of Engineering, Biotechnology and Applied Biochemistry—LIBBA, Department of Chemical Engineering, Faculty of Engineering and Science, La Frontera University, Temuco, Chile; 8 Department of Biological and Chemical Sciences, Faculty of Natural Resources, Catholic University of Temuco, Temuco, Chile; 9 Nucleus of Environmental Sciences, Faculty of Natural Resources, Catholic University of Temuco, Temuco, Chile; 10 Estudiante Magister en Estadística, Instituto de Estadística, Facultad de Ciencias, Pontificia Universidad Católica de Valparaíso, Valparaíso, Chile; 11 Department of Botany and Microbiology, College of Science, King Saud University, Riyadh, Saudi Arabia; National Institute of Biologicals (NIB), Ministry of Health & Family Welfare, Government of India, INDIA

## Abstract

*Salmonella typhimurium*, a Gram-negative bacterium, is a significant cause of gastroenteritis worldwide, with outbreaks occurring in diverse regions. Despite its global impact, there is currently no vaccine for human use against this pathogen. Complicating treatment efforts, *S. typhimurium* has exhibited resistance to multiple antibiotics, posing challenges to effectively managing infections. Given its prevalence and unresolved antibiotic resistance–associated global health burden, urgent attention is required to develop a genuinely effective vaccine. Using the *S. typhimurium* complete proteome data, vaccinomics-assisted immunoinformatics techniques were employed in the current investigation to find possible vaccine candidates. Candidate proteins were identified based on essentiality, lack of homology with the human proteome, and absence from the gut microbiome. Using a reverse vaccinology methodology, four antigenic outer membrane proteins were ranked in order of priority for lead epitope prediction. To boost immune responses against the intended vaccination, lead B and T-cell epitopes were coupled with appropriate linker and adjuvant peptide sequences to create multiepitope-based chimeric vaccines. The ST-MEVC construct was ranked according to several immunological, physicochemical, and immune receptor docking scores. Immune simulation predicted a strong immunogenic response for the proposed vaccine formulation. Molecular dynamics simulations analysis confirmed stable molecular interactions between the primary vaccine construct and the host receptors. The ST-MEVC construct’s feasible cloning potential within the *E. coli* expression system was anticipated by in silico restriction and cloning studies. The proposed vaccine design is expected to elicit more robust immune responses against *S. typhimurium* infections and will be safer, more efficacious, and more promising for investigation using in vitro/in vivo assays.

## 1. Introduction

*Salmonella typhimurium* (*S. typhimurium*) or *Salmonella enterica* serotype typhimurium is a member of the *Enterobacteriaceae* family. It is a Gram-negative, motile, rod-shaped bacterium [1], frequently linked to intestinal cell invasion and inflammation in the host. Since *S. typhimurium* is facultatively anaerobic [2], it can thrive both with and without oxygen. It is among the most frequent causes of foodborne illnesses in both humans and animals [3], such as salmonellosis. Symptoms associated with salmonellosis include diarrhea, abdominal pain, fever, vomiting, nausea, headache, muscle pain, and dehydration. *S. typhimurium* infections are widespread globally, impacting both high-income and low-income countries [4]. *Salmonella* infections are more severe in the regions where hygiene standards are insufficient [5]. Salmonellosis can quickly spread from person to person and is very common [6]. It is primarily transferred through poultry, eggs, meat, dairy products, and crops that have become contaminated during processing or handling [7]. According to the World Health Organization (WHO), over 2 billion people are affected by diarrheal diseases annually, with *S. typhimurium* contributing significantly to foodborne illnesses. [8].

*S. typhimurium* infections cause substantial morbidity and mortality. WHO estimates that non-typhoidal *Salmonella*, including *S. typhimurium*, is responsible for approximately 93.8 million gastroenteritis cases and over 150,000 deaths annually [9]. Similarly, studies have shown that invasive non-typhoidal *Salmonella*, including *S. typhimurium*, contributes to high mortality rates, particularly in vulnerable populations in sub-Saharan Africa and Southeast Asia [10]. Among the 173 serotypes examined, *S. typhimurium* accounts for 36.07% of strains, followed by *S. enteritidis* 15.17%, and *S. london* 6.05% [11]. Symptoms typically appear between 6 and 72 hours after infection and persist for 4–7 days [12].

*S. typhimurium* has multiple virulence factors, such as adhesions, type III secretion systems, and toxins that help it colonize and avoid the host’s immunological response [13]. While most individuals recover without consuming antibiotics, those with severe infections and underlying illnesses that weaken their immune systems should consider receiving antibiotics. Antibiotics are efficacious in treating *Salmonella* infections and decreasing the duration of the disease in an infected person [14]. However, the emergence of antibiotic-resistant strains, including those resistant to trimethoprim, sulfamethoxazole, ampicillin, fluoroquinolones, gentamicin, tetracycline, azithromycin, and meropenem, exacerbates the public health burden and complicates treatment [15]. This resistance is a growing concern in many regions and significantly complicates treatment options, highlighting the need for updated surveillance and interventions [16]. Drug resistance in *S. typhimurium* is caused by drug extrusion through active efflux pumps, overexpression of enzymes that deactivate and change medicines, reduction in cellular permeability, and target modification due to mutations [17]. The development of resistance in *Salmonella* strains against several drugs and the continuous high incidence of the disease suggest that salmonellosis is a permanent and unsolved global health burden [18].

Numerous vaccines of either killed or live-attenuated bacteria have been tested through different clinical phases; nevertheless, no commercial vaccine is currently available against *Salmonella*. Research has shown varied levels of immunogenicity despite efforts to produce robust and long-lasting immune responses [19]. There is a dire need to optimize vaccine development techniques to combat deadly multidrug-resistant pathogens like *S. typhimurium.* The accessibility of large-scale genomic and proteomic data and advancements in bioinformatics and immunoinformatics have intrigued the development of more effective and safe vaccines against targeted infections. The current work identified possible vaccine-candidate proteins in the *S. typhimurium* proteome using vaccinomics-based immunoinformatics tools. Subsequently, reverse vaccinology methodologies were employed to generate a vaccine design based on multiple epitopes. Further evaluations of the vaccine’s thermodynamic stability, ability to bind to human immunological receptors, and virtual cloning in a bacterial expression system were conducted. Immunological simulation analysis was used to examine the efficacy of the developed vaccine [20].

Reverse vaccinology has been shown to accelerate the design of effective vaccines by identifying epitopes with high immunogenic potential, making it a valuable approach for vaccine development [21,22]. Recent developments in large-scale computational biology, including multi-level network embedding and hybrid multiscale module–based prediction frameworks, further support the use of advanced bioinformatic pipelines for rapid identification and prioritization of biological targets [23–25]. In the present study, we employed an in silico immunoinformatics approach, which refers to a layered computational pipeline integrating multiple analyses for rational vaccine design. This strategy involves several steps, including proteome retrieval, subtractive proteomics, epitope prediction, multi-epitope assembly, structural modeling, docking, immune simulation, and molecular dynamics. Together, these steps create a systematic framework for designing immunogenic and safe vaccine candidates.

**Fig 1:**
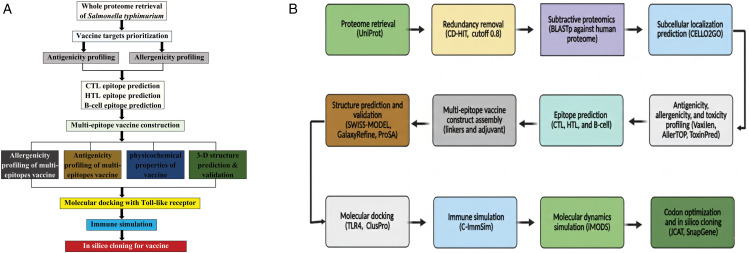
A. Complete step-by-step process of the methodology employed in the design of the vaccine against *S. typhimurium.* B. Pipeline of the in silico immunoinformatics approach for multi-epitope vaccine design against *S. typhimurium*.

An overview of the complete methodological workflow is illustrated in Fig 1A. The complete procedure can be separated into two primary components: subtractive proteomics forms the basis of the first part, while structural vaccinology methods were used in the second to map the immunogenic epitopes in the chosen proteins. Finally, these epitopes were combined to create ST-MEVC, confirmed by immunological simulation, molecular docking, and in silico cloning.

## 2. Materials and methods

The workflow begins with proteome retrieval from UniProt, followed by redundancy removal using CD-HIT and subtractive proteomics with BLASTp against the human proteome. Candidate proteins were screened for subcellular localization (CELLO2GO) and further filtered for antigenicity, allergenicity, and toxicity (VaxiJen, AlgPred, and ToxinPred). Validated proteins were subjected to B-cell, cytotoxic T lymphocyte (CTL), and helper T lymphocyte (HTL) epitope prediction. Selected epitopes were linked with suitable adjuvants and linkers to generate the multi-epitope vaccine construct. The construct was then modeled and validated (Swiss-Model, GalaxyRefine, ProSA), followed by molecular docking with TLR4 (ClusPro). The immune response was simulated in silico (C-ImmSim), molecular dynamics were analyzed (iMODS), and codon optimization with in silico cloning was performed (JCAT, SnapGene) to assess expression feasibility in *E. coli*.

### 2.1. Proteome retrieval and screening for non-redundant proteins

The complete proteome data of the *S. typhimurium* was retrieved from the UniProt knowledgebase [26], which is accessible with the accession number UP000001014. In order to remove the redundant and paralogous protein entries, the entire set of *S. typhimurium* proteins was submitted to the CD-HIT server [27]. A cutoff value of 0.8 was selected, and the resulting non-paralogous protein sequences were retained for downstream analysis [28].

### 2.2. Removal of human homologues and obtaining pathogen-specific proteins

Using the NCBI BLASTp tool, the non-paralogous *S. typhimurium* proteins were aligned to the entire human proteome (Taxonomy ID 9606), with the threshold values of E-value cut off value of 10^−4^, bit score ≤100, percent identity ≤35, and query coverage ≤35 [29], to exclude proteins homologous to human (host) proteins using the subtractive proteomics technique. Only the proteins specific to the pathogen were kept for further analysis.

### 2.3. Subcellular localization of proteins

The CELLO2GO v.2.5 web server was used to locate non-homologous pathogen-specific proteins’ subcellular localization. This server combines the CELLO localization prediction tool and the BLAST homology search tool to find out the targeting of proteins into different compartments and organelles of the cell [30]. The pathogen-specific proteins localized to extracellular spaces and outer membranes were further selected for the prediction of candidate vaccines [31].

### 2.4. Prioritizing potential vaccine candidates

Extracellular proteins and those localized to outer membranes were evaluated to determine their antigenicity, allergenicity, and toxicity. The antigenicity of the chosen proteins was checked on the VaxiJen v2.0 web server [32], with 0.5 selected as the threshold value [33]. VaxiJen v2.0 has been shown to achieve an overall accuracy of ~87% with AUC > 0.8 based on leave-one—out cross-validation [34]. The antigenic chosen proteins were further evaluated for allergenicity on the AlgPred2 web tool [35]. Upon its development, AlgPred2 achieved an accuracy of 87.05 in 5-fold cross-validation on a benchmark dataset of allergenic and non-allergenic proteins [36]. The AllerTOP v2.0 predicts allergenicity using amino acid E-descriptors (z-scores reflecting physicochemical properties like hydrophobicity, size, polarity, helix-forming propensity, etc.). Sequences are transformed into uniform-length vectors through auto cross-covariance (ACC) transformation, which converts variable-length sequences into feature matrices based on residue correlations. These transformed vectors are then classified by machine learning models to distinguish allergens from non-allergens. The antigenic non-allergenic proteins were tested for their toxicity on the ToxinPred server [37]. ToxinPred predicts peptide toxicity based on quantitative structure–activity relationship (QSAR) descriptors (e.g., hydrophobicity, hydropathicity, charge, amino acid composition, and dipeptide composition). These features capture how peptide physicochemical properties relate to toxicity. The tool uses support vector machine (SVM) classifiers trained on experimentally validated toxic and non-toxic peptides to discriminate between the two classes [38]. The ToxinPred achieved an accuracy of ~97% in distinguishing toxic vs non-toxic peptides [39]. To reduce false positives, we applied a consensus strategy: epitopes were retained only if predicted consistently across ≥2 servers. Thresholds were set as follows: VaxiJen ≥ 0.4, NetCTL ≥ 0.75, ABCPred ≥ 0.51, AllerTOP cutoff = 0.5, and ToxinPred classified as non-toxic. The antigenic, non-allergenic, and non-toxic selected pathogen-specific proteins were considered for further analysis of the B and T-cell epitope prediction. These categories of proteins were prioritized because antigens that are non-allergenic and non-toxic are more likely to be safe and effective vaccine candidates. Antigenic proteins stimulate protective immune responses, while exclusion of allergenic and toxic proteins reduces the risk of adverse reactions. Thus, selecting pathogen-specific proteins with these characteristics ensures a balance between immunogenicity and safety, which is a fundamental principle in rational vaccine design.

### 2.5. Prediction of T and B-cell epitopes

The cytotoxic T-cell (CTL), B-cell, and helper T lymphocyte (HTL) epitopes’ predictions in the prioritized *Salmonella* proteins were made using the NETCTL 1.2 web tool [40], the ABCPred web server [41], and the IEDB database MHC II module [42], respectively. The ABCPred predicts B-cell epitopes using an artificial neural network (ANN) based model trained on fixed-length epitope datasets, enabling recognition of patterns in antigen sequences [43]. The NetCTL integrates peptide binding, proteasomal cleavage, and TAP transport predictions through support vector machine (SVM)–based classifiers, providing accurate CTL epitope predictions [44]. IEDB epitope prediction tools have been validated with AUC values between “0.70-0.85” depending on allele and dataset [45]. A threshold of 0.75 was applied for CTL epitope prediction, whereas a threshold of 0.51 was used for B-cell epitope prediction to keep specificity at 75%. It was predicted that HTL epitopes would be present in five distinct human HLA alleles (DRB1*01:01, DRB1*01:02, DRB1*01:03, DRB1*01:04, and DRB1*01:05). The IC_50_ value was used to quantify the peptide’s receptor binding affinity. The binding affinities were found to be high, moderate, or low when the IC_50_ scores were less than 50 nM, 500 nM, or 5000 nM, respectively. Epitopes with IC_50_ values less than 50 nM were given priority while developing immunogenic constructs [46]. The shortlisted CTL, HTL, and B-cell epitopes were mapped onto the 3D structures of their respective proteins using UCSF Chimera (version 1.16). Distinct color codes were applied to each epitope category to facilitate visualization of their spatial distribution and surface accessibility.

### 2.6. Vaccine construction

We used AAY and GPGPG linkers to forecast and link CTL, HTL, and B-cell epitopes. The CTL epitopes were linked using AAY linkers, and the last CTL epitope was linked to HTL and B-Cell epitopes using GPGPG linkers. Human beta-defensin 3 was incorporated as an adjuvant to enhance immunogenicity while maintaining safety. These linkers enhanced epitope processing and presentation via TAP transport and increased the stability of the immunogens. The epitope presentation was enhanced by the GPGPG linker, which also stimulated HTL responses and preserved the conformation-dependent immunogenicity of helper cells and antibody epitopes [47,48].

### 2.7. Validation of 3D structures with homology modeling

The three-dimensional structures of the multi-epitope vaccine complexes were predicted using the Swiss-Model server [49]. The GalaxyRefine web server was utilized to improve the anticipated vaccine structures [50]. Using ERRAT, PROCHECK, and ProSA web tools, the accuracy of the vaccine constructions’ refined 3D structures was further validated [51].

### 2.8. Physicochemical properties

ExPASy’s ProtParam was utilized to predict the vaccine’s physicochemical characteristics. ProtParam also calculated a peptide vaccine sequence’s stability, theoretical PI, aliphatic index, molecular weight, GRAVY (grand average of hydropathicity), and amino acid content [20].

### 2.9. TLR4 and vaccine constructs’ molecular docking

The Cluspro web server (Stony Brook, New York, USA) was used for molecular docking of the vaccine construct with human TLR4 to find out how ST-MEVC interacts with the immunological receptor. We evaluated whether the final vaccine construct could dock to the TLR4 homodimer (PDB ID: 3FXI). To continue this investigation, the MECV-TLR4 complex that exhibited the lowest energy score during docking was chosen [26].

### 2.10. Optimization of codons and cloning of the vaccine construct

The ultimate vaccine design employed codon optimization and in silico restriction cloning. To enhance the expression of the cloned sequence in the *E. coli* expression system, the codons of the peptide vaccine sequence were improved using the Java Codon Adaptation method (JCAT) [27]. JCAT examines the CAI (codon adaptation index) and the GC content percentage to identify the cloned vaccine sequence’s highest expression potential. To acquire the best results in transcription and translation, GC content was maintained between 30% and 70%, and a CAI value of 1. The ST-MEVC expression in *E. coli* was improved by employing restriction cloning via Snapgene software [29]. After completing the restriction cloning, the *E. coli* plasmid pET28a TIAL1 acquired from the Addgene website was utilized [30].

### 2.11. Immune simulation

The C-ImmSim server [31] was utilized to conduct computational immunological simulation on the created vaccine design to evaluate its immunogenicity [32]. The server offers data on cellular and humoral reactions to antigens and uses several machine learning methods to forecast probable stimulation of the host’s immune system [35]. According to the standard clinical protocol, a four-week interval was observed between consecutive vaccine doses [37]. In this investigation, as previously done, we replicated the established technique to conduct an immunological simulation of the intended vaccine formulation. The simulation parameters were set to their default values for 1 hour, 84 hours, and 168 hours. For the simulation, we chose the human host leukocyte antigens HLA-A*0101, HLA-A*0201, HLA-B*0702, HLA-B*3901, HLA-DRB1*0101, and HLA-DRB1*0401. We ran the simulation for 1000 steps.

### 2.12. Molecular dynamics simulation and trajectory analysis

Molecular dynamics (MD) simulation was conducted on the vaccine-TLR4 complex with the most favorable docking analysis results. The MD simulations, energy minimization, and the flexibility of protein estimate were performed on the iMODS server [40]. The server can illustrate the complex dynamics of macromolecules by utilizing an advanced arrow representation, the base of which is an affine model, detect possible conformational changes, identify elastic network potentials, and model resolution using several coarse-grained atomic representations. The server analyzed the structural dynamics of proteins and their interactions with other molecules and ligands. It provides data on elastic networks, variance, B-factor (mobility profiles), eigenvalues, covariance maps, and deformability [41]. We used the iMODS service to upload the docked PDB file of the vaccine-TLR4 complex and got results with all the default parameters.

To further validate the stability of the ST-MEVC–TLR4 complex, a 100 ns molecular dynamics (MD) simulation was conducted. The docked complex was subjected to all-atom simulation using standard force fields, and the trajectory was analyzed for conformational stability. Root Mean Square Deviation (RMSD) was computed to assess global stability, while Root Mean Square Fluctuation (RMSF) quantified the flexibility of individual residues. Additionally, a Dynamic Cross-Correlation Matrix (DCCM) was constructed to evaluate correlated and anti-correlated motions between residues. Principal Component Analysis (PCA) was performed on the trajectory to identify dominant motions and conformational states of the complex. All simulation trajectories were visualized and analyzed using appropriate visualization tools, and figures were generated to illustrate dynamic behavior.

## 3. Results

### 3.1. Proteome retrieval and filtration of non-paralogous proteins

The entire proteome of *S. typhimurium*, identified by the UniProt ID UP000001014, was downloaded from the UniProt database. The pathogen’s proteome contains 4533 proteins. We used the subtractive proteomics approach to find candidate proteins, identifying which proteins would be the most suitable targets for a vaccine and establishing an efficient vaccination ensemble.

The complete set of 4,533 proteins was submitted to the CD-HIT server. After using a threshold value of 0.8 to exclude paralogous sequences, the analysis revealed that 2085 proteins out of 4533 were non-paralogous. The next step was to compare the *S. typhimurium* non-paralogous sequences to a human reference proteome using taxonomy ID 9606. This step eliminated proteins homologous to the human proteome.

### 3.2. Subcellular localization

Understanding cellular localization is essential to identifying proteins suitable for vaccine targeting. The Psortb tool was employed to analyze the complete set of 2,085 *S. typhimurium* proteins, categorizing them by their cellular location, including the cytoplasmic, periplasmic, extracellular, and membrane-associated regions (both internal and external) (Table 1). Among these, four proteins (UniProt IDs: Q8ZNX5, O31041, Q8ZRG3, and P0A2K1) localized to extracellular and outer membrane regions were prioritized due to their higher antigenicity scores of 0.4594, 0.5236, 0.5139, and 0.4218, respectively (Table 2). These antigenicity predictions from VaxiJen were further validated using ANTIGENpro and IEDB’s antigenicity prediction tool, both of which corroborated the classification of these proteins as antigenic.

**Table 1:** Prediction of the subcellular location of the selected proteins.

S. No	Cellular Localization	Number of Proteins
**1**	Outer Membrane	41
**2**	Extracellular	25
**3**	Periplasmic	71
**4**	Cytoplasmic Membrane	529
**5**	Cytoplasmic	1392

**Table 2:** Details of the final shortlisted vaccine targets, including name, length, and UniProt ID, used in the vaccine design process.

S.No	UniProt ID	Name of protein	Length	Antigenicity	Allergenicity	Toxicity	Antigenic Pro
**1**	Q8ZNX5	Phage integrase Protein	302	Antigenic	Non-allergenic	Non-toxic	0.191613
**2**	O31041	Propanediol dehydratase medium subunit	227	Antigenic	Non-allergenic	Non-toxic	0.856644
**3**	Q8ZRG3	CytochromeBD2 subunit I	474	Antigenic	Non-allergenic	Non-toxic	0.091604
**4**	P0A2K1	Tryptophan synthase beta chain	403	Antigenic	Non-allergenic	Non-toxic	0.345586

The four shortlisted proteins (Q8ZNX5, O31041, Q8ZRG3, and P0A2K1) were subsequently employed for downstream immunoinformatics analysis. Specifically, these proteins served as input for CTL and HTL epitope prediction (Tables 3 and 4), B-cell epitope mapping (Table 5), and ultimately guided the construction of the multi-epitope vaccine chimera (Section 3.6). This approach is consistent with previously reported reverse vaccinology-based pipelines for vaccine development [52,53].

**Table 3:** List of CTL epitopes chosen for the vaccine design. The binding affinity values are reported as IC_50_ values in nanomolar (nM).

Uniprot ID	Peptide sequence	Affinity of MHC Binding	Affinity of rescale binding	C-terminal affinity for cleavage	Transport affinity	Combined score	MHC-I binding
**Q8ZNX5**	TSENTTKLY HTSENTTKL	0.6777 0.1771	2.8774 0.7520	0.9411 0.9782	2.7770 0.8080	3.1574 0.9392	Yes Yes
**O31041**	SSDVAFVAV QIGKNAARY	0.2872 0.1567	1.2192 0.6652	0.9720 0.9730	0.3330 3.0350	1.3817 0.9629	Yes Yes
**Q8ZRG3**	YSLVFGVGY DMEAERTRY	0.3499 0.2968	1.4856 1.2600	0.2795 0.9399	3.0100 2.4410	1.6780 1.5230	Yes Yes
**P0A2K1**	TAGTRTTLY ITDDEALEA	0.3918 0.3478	1.6634 1.4768	0.9654 0.6260	2.8020 −0.6560	1.9483 1.5379	Yes Yes

**Table 4:** List of selected HTL epitopes for vaccine design.

Protein ID	Allele	Start	End	Peptide Sequence	Methods	Percentile Rank
**Q8ZNX5**	HLA- DRB4*01:01	5	39	SENTTKLYLDERDNK	Consensus (comb.lib./smm/nn)	1.3
	HLA- DRB3*01:01	41	62	ALIEKLKGTDSFTVS		2.5
**O31041**	HLA- DRB3*02:02	4	29	KYVVTGKNPQELRVA	NetMHCIIpan	0.49
	HLA- DRB4*01:01	31	52	DEVIIAVGPAFGLAQ	Consensus (comb.lib./smm/nn)	1.4
**Q8ZRG3**	HLA- DRB1*03:01	6	22	FWSFRLMVGMGVLMI	Consensus (smm/nn/sturniolo)	1.2
	HLA- DRB1*03:01	2	4	RLLHMSVAAFLSSAM		1.2
**P0A2K1**	HLA- DRB1*03:01	5	33	HGRVGIYFGMKAPMM	Consensus (smm/nn/sturniolo)	
	HLA- DRB1*03:01	1	2	TTLLNPYFGEFGGMY		

**Table 5:** List of B-cell epitopes used for the vaccine formulation.

Protein ID	Type of Epitope	Sequence	Position	Length AA	Score
**Q8ZNX5**	B-cell	SAGIRKNSPTGNIHPDGL	212	18	0.87
**O31041**	B-cell	RYAKRESPQPVPTLNDQM	174	18	0.84
**Q8ZRG3**	B-cell	SFPYRLLHMSVAAFLSSA	178	18	0.81
**P0A2K1**	B-cell	TKCQNITAGTRTTLYLKR	60	18	0.91

### 3.3. CTL and HTL epitopes prediction

T-lymphocytes are essential for initiating an immune response to specific pathogens [51]. These T-cell epitopes can activate either CD4 helper T-cells or CD8 cytotoxic T-cells [54]. We also anticipated T-cell epitopes (CTL) to stimulate the immunogenic response against *S. typhimurium*. NetCTL 1.2 predicted a total of 29 epitopes for selected proteins. However, only eight of those epitopes met the requirements for antigenicity, non-allergenic nature, and MHC binding score. The determined epitopes are presented in Tables 3 and 4. Helper T-cells are also essential components necessary for the correct induction of acquired or adaptive immunity. HTL epitopes assist the B-cell in producing and secreting antibodies. Moreover, it helps the CTL destroy harmful cells. These effectors need to be activated and displayed on the surface of APCs to initiate a specific immune response, after which they can transform into the appropriate type of HTL. We expected helper T-cell epitopes to facilitate the activation of a robust immunological reaction by B and T-cell epitopes, given the importance of HTL epitopes in this setting. To predict HTL epitopes, the IEDB MHC-II server used seven human HLAs: HLA-DRB4*01:01, HLA-DRB3*01:01, HLA-DRB1*15:01, HLA-DRB5*01:01, HLA-DRB5*01:01, and HLA-DRB1*07:01. Details of the HTL epitopes are provided in Table 3. The four proteins were analyzed for their allergenicity, antigenicity, and low percentile rank, and eight HTL epitopes were chosen.

### 3.4. B-cell epitopes prediction

B-cell receptors are membrane-bound globular proteins that enable B-cells to recognize antigens present in soluble form. Upon activation, B-cells secrete antibodies, which are soluble forms of immunoglobulins. Antibodies facilitate adaptive humoral immunity. The binding of antibodies to their respective targets induces a wide range of effects. 3D structural mapping confirmed that the predicted B-cell epitopes were surface-exposed and accessible. ABCpred developed four B-cell epitopes, each consisting of 18 amino acids with a score of > 0.90. B-cell epitopes that were evaluated as non-allergic and non-toxic peptides, along with the highest performance, were chosen. The best antigenic, non-toxic, and non-allergic B-cell epitopes selected from the Salmonella proteins extracted from ST-MEVC are presented in Table 5. In addition to the sequence-based analysis, all predicted CTL, HTL, and B-cell epitopes were mapped onto the 3D structures of their respective proteins using UCSF Chimera. Distinct color codes were applied to each epitope category, and the resulting structural visualizations confirmed that the majority of epitopes were surface-exposed and accessible (S1–S3 Figs).

### 3.5. Multi-epitope immunogen construction

**Fig 2:**
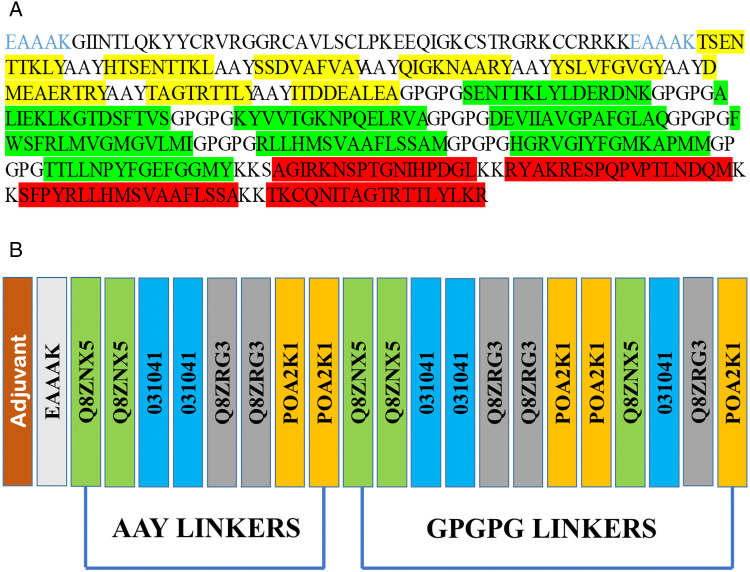
A. The ST-MEVC design is depicted as a sequence, with adjuvants represented in black, epitopes in yellow, green, and red, and linkers in black and blue. B. The structural configuration of the several epitopes joined by linkers. The adjuvant is joined at the N-terminal, and a blue line represents the linkers for AAY, GPGPG, and EAAK.

The primary goal of developing a multi-epitope chimera vaccine was to elicit a robust immune response against *S. typhimurium* infections. To ensure high immunogenicity, the vaccine was designed using prioritized HTL, B-cell epitopes, and CTL epitopes. The vaccines require antigenic epitopes, adjuvants, and linkers specific to these epitopes to generate an adequate response. In this study, beta-defensin 3

(GIINTLQKYYCRVRGGRCAVLSCLPKEEQIGKCSTRGRKCCRRKK) was applied as an N-terminal adjuvant to enhance the efficacy of the ST-MEVC vaccine while minimizing the potential side effects [55]. The designed vaccine comprised eight CTL epitopes, eight HTL epitopes, and four B-cell epitopes. Furthermore, GPGPG linkers were used to connect HTL epitopes to B-cells, while KK linkers joined the B-cells together. The AAY linkers were also employed to CTL epitopes, while the adjuvant was fused to the CTL epitopes using EAAAL linkers. This arrangement ensures that each epitope in the multi-epitope vaccine can independently trigger an immune response while also contributing to the overall immunogenicity of the vaccine (Figs 2A and 2B).

### 3.6. 3D structure prediction and validation

**Fig 3:**
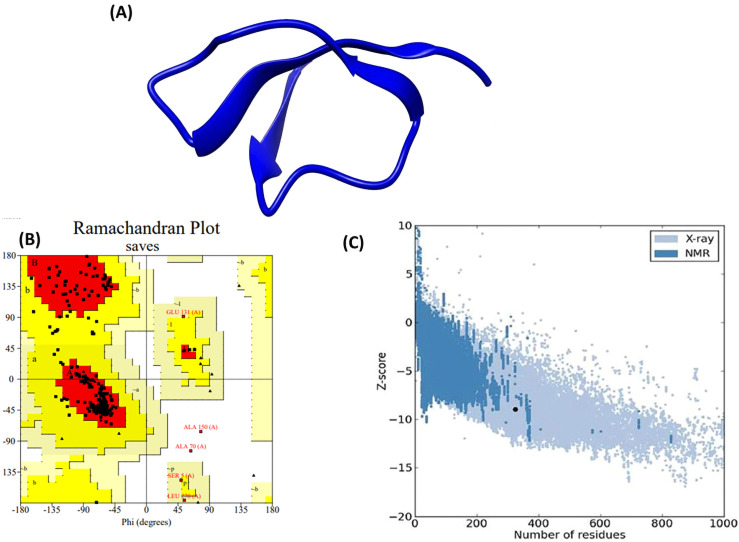
Prediction and validation of the tertiary structure of ST-MEVC. **(A)** The Swiss model of the vaccine’s three-dimensional structure. **(B)** ST-MEVC structure validation using ProSA-web, yielding a Z-score of −4.78. **(C)** PROCHECK’s Ramachandran plot explores 90.0% of the residues in the plot’s preferred area.

A stable and functional 3D structure of the vaccine is essential to study the interaction between the vaccine and immunological receptor proteins. To achieve this, homology modeling of the vaccine construct was performed using the Swiss Model server, as shown in Fig 3A. The Swiss-Model server reported a template (sequence) identity of 63.64% and a GMQE score of 0.05, providing an initial estimate of model reliability. Although the GMQE score was relatively low, subsequent refinement using GalaxyRefine and validation through stereochemical analyses improved the structural plausibility of the model. This was followed by refinement through the Galaxy web server and structure variation analysis (Fig 3B). GalaxyRefine generated five refined models, which were compared with the initial structure. As summarized in Table 6, all refined models exhibited minor deviations from the initial model (RMSD ~0.32–0.35 Å), while showing improvements in stereochemical quality. Notably, the refined models demonstrated higher percentages of Ramachandran favored residues (up to 94.0%) and reduced poor rotamers, confirming enhancement in structural plausibility.

**Table 6:** Structural comparison of the initial and refined vaccine construct models generated by GalaxyRefine.

Model	GDT-HA	RMSD	MolProbity	Clash score	Poor rotamers	Rama favored
Initial	1.0000	0.000	1.919	6.2	1.0	89.1
MODEL 1	0.9794	0.332	2.264	15.6	1.4	92.5
MODEL 2	0.9800	0.320	2.137	14.7	1.0	92.7
MODEL 3	0.9755	0.338	2.107	15.1	0.3	93.5
MODEL 4	0.9697	0.347	2.269	13.4	2.0	94.0
MODEL 5	0.9774	0.319	2.208	13.9	1.4	92.7

The Ramachandran plot for the vaccine construct showed that 90% of the beta-defensin construct was located in the preferred region of the plot (Fig 3C). The ERRAT tool applied further confirmed the quality of the refined vaccine design, with an overall quality factor of 90%. Furthermore, the Z-score for the vaccine construct was determined to be −5.94 using the ProSA-web server. These collective results from RAMPAGE, ERRAT, and ProSA-web validated the high quality of the modeled 3D vaccine structure.

### 3.7. Determination of physicochemical characteristics of vaccine construct

Identifying the physicochemical properties is essential for assessing the stability of the vaccine construct for further experimentation. The ProtParam tool was used to extract the vaccine construct’s physicochemical characteristics, including molecular weight, half-life, instability index, etc, of the vaccine construct. The results showed the molecular weight of the final protein was determined to be 33.71 kDa, with a GRAVY score of −0.240. The half-life for the vaccine construct was determined to be > 1 h in mammalian reticulocytes, 30 min in yeast cells, and >10 h in *E. coli*. Additionally, the instability index was 28.16, while the aliphatic index was 79.94, indicating that the construct is exceptionally stable. The antigenicity score of the designed vaccine construct was calculated to be 0.8594. All these physicochemical properties predict that the vaccine construct has high stability and is a remarkable vaccine candidate.

### 3.8. Examination of Immunogenic Construct Interaction with Toll-Like Receptors

**Fig 4:**
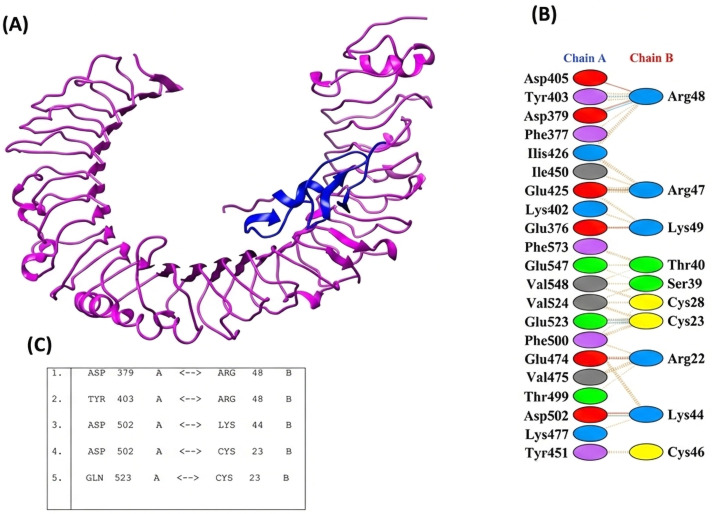
3D representation of the docking sample. (A) Three-dimensional depiction of the ST-MEVC docking complex utilizing human TLR4. (B, C) SS-MEVC chain A and TLR4 chain B molecules’ molecular connections.

The interaction analysis between the proposed immunogen and human TLR receptors provides detailed insights into the binding affinity required for the complex formation. A total of 30 clusters for vaccine receptors were produced from vaccination and receptor complexes using the ClusPro website (https://cluspro.bu.edu). Among these 30, the most suitable docked model was selected (Fig 4) using PyMOL, Version 1.7.1. The final model chosen exhibited the lowest energy score of −672.1, indicating a highly stable binding conformation.

### 3.9. Codon optimization and in silico cloning

**Fig 5:**
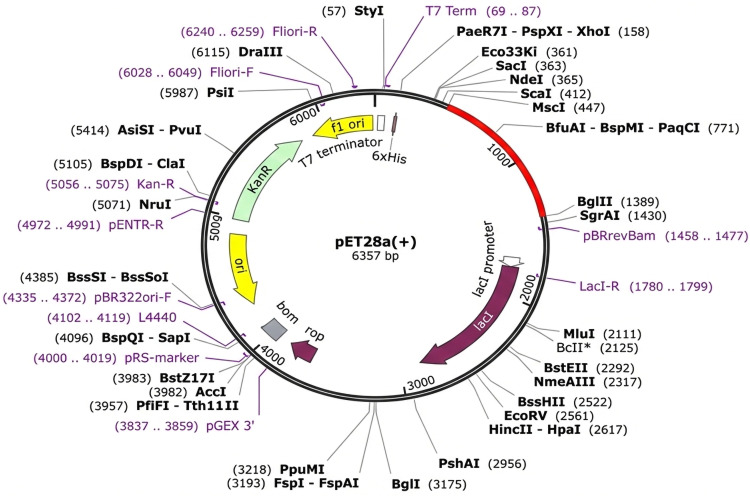
The previous vaccine design was replicated into the pET28a (+) expression vector via in silico restriction cloning. The red area represents the vaccine insert, while the black circle shows the vector.

The expression potential of the proposed construct was evaluated using JCAT. The JCAT results predicted that the reverse-translated and optimized sequence of the vaccine construct was 987 nucleotides long, with a GC content of 54.44% and a CAI score of 0.92. The results signify the optimal range of favorable expression of the vaccine construct in the *E. coli* K12 system. Finally, the recombinant plasmid sequence was constructed by inserting the modified vaccine sequence into the pET2 (+) plasmid vector using SnapGene software. The *Msc*I and *Bg*III restriction enzymes were selected to cleave the vector and construct the vaccine from 5 ‘to 3 ‘. Fig 5 shows the map of the constructed pET28a (+) expression vector inserted into the *E. coli* strain K12.

### 3.10. Immune simulation

**Fig 6:**
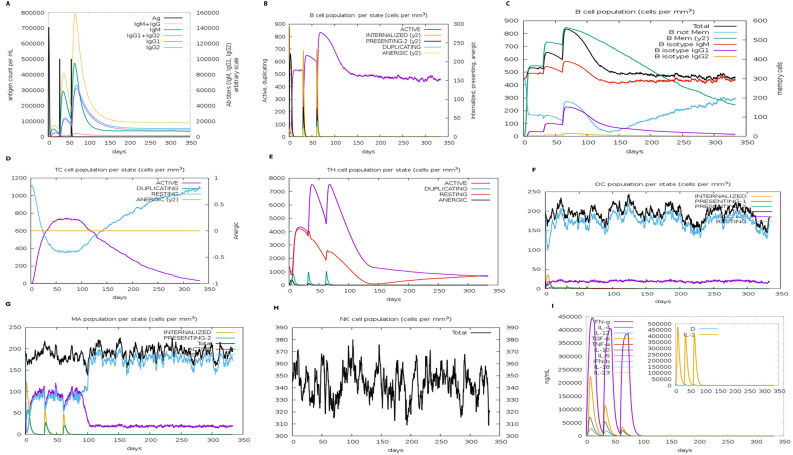
V chimeric peptide for in silico immunological activation, as forecasted by C-ImmSim Server A and B. Antigen levels fall sharply as immunoglobulin antibody levels and B-cell populations rise(C). The maturation of B-cells in response to antigen stimulation. (D, E) The increase in the number of T-cytotoxic and T-helper cells is caused by repeated exposure to antigens. The vaccination phase was characterized by the rise in the population of macrophages, dendritic cells, and natural killer cells (F, G, H), (I), as a result of higher cytokine concentrations caused by repeated antigen exposure.

Immune simulation performed through the C-ImmSim server showed a significant increase in primary and secondary immune responses against the proposed vaccine construct (Fig 6). Enhanced levels of IgM, IgG1 + IgG2, and IgM + IgG2 immunoglobulin antibodies were observed after the administration of the vaccine, confirming the proliferation of immune responses (Fig 6A). The proliferation of a strong immune response significantly decreases the antigen count per mL, as illustrated in Fig 6A. Repeated exposure to the antigenic vaccine resulted in the development of humoral immune memory, as an increase in the B-cell population was evident in Figs 6B and 6C. Further, it was observed that during secondary and tertiary responses, the population of CTL and HTL increases significantly with a substantial decrease in antigen population (Figs 6D and 6E). Moreover, it was evident that administering the vaccine construct led to the activation and preservation of significant quantities of macrophages, natural killer cells, and dendritic cells, as portrayed in Fig 6F-6H. The vaccine construct also increased the levels of cytokines and interleukins, including IFN-γ and TGF-β, as presented in Fig 6I. These findings confirm that the vaccine construct provides strong protection against *S. typhimurium.*

### 3.11. Dynamic simulation of molecules

**Fig 7:**
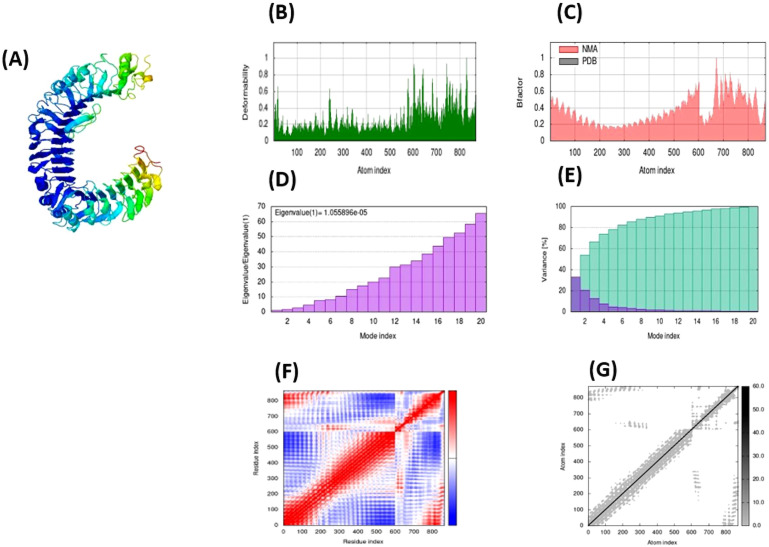
The outcomes of the TLR4 complex and vaccine construct molecular dynamics simulations produced by the iMODS server. NMA mobility in **(A)**, deformability in **(B)**, and the B-factor, which represents an averaged RMS, in **(C)**. Eigenvalues in **(D) (E)** Colored bars display the cumulative (green) and individual (purple) variances; **(F)** The covariance matrix displays the paired residues correlated (blue), uncorrelated (white), and anti-correlated (red) movements. **(G)** In the model of elastic networks, stiffer parts are indicated by grey areas.

Computational (MD) simulations primarily evaluate the dynamic behavior of the docked molecules over time. MD simulations help to determine accurate binding conformations, protein folding patterns, and the stability of the molecular interactions inside the cellular environment. Therefore, the MD simulation technique was employed to evaluate protein stability, energy minimization in the vaccine construct-TLR4 complex, and vaccine movement using iMODS. Fig 7A displays Normal mode analysis (NMA) findings using the (MD) simulation for the vaccine construct-TLR4 docked complex. To simulate potential changes, the input structure was gradually altered by manipulating its lowest modes, and the target structure’s root mean square deviation (RMSD) was reduced by considering both the local and overall alignment of the structures. The cumulative atomic displacements over all residue modes at each atomic site indicate main-chain deformability. The peaks on the complex’s deformability graph indicate the protein’s pliable areas. The chain’s flexible components (hinges/linkers) frequently exhibit greater values than the rigid sections of the central chain residues. The deformability graph (Fig 7B) displays the peaks of the graphs, which indicate the distorted portions of the protein. The B-factor generated by the NMA determines the relative amplitude of the atomic displacements of the molecular complex in its equilibrium conformation. The link between the PDB scores and the mobility of the docked complex NMA is shown by the B-factor graph in Fig 7C, which shows the average RMSD. The eigenvalue of each normal mode represents the motion stiffness. The energy required to alter the shape of a structure is strongly linked to this value. A reduction in the eigenvalue indicates an enhancement in the alpha carbon atoms’ deformation efficiency. The eigenvalue of the TLR4 complex and vaccine construct, 1.055896e-05 (Fig 7D), suggests the complex’s stability level. Each mode of the complex is linked to the variance graph, which exhibits an inverse relationship with the eigenvalue in Fig 7E. The graph displays individual (purple) and cumulative (green) variances. The covariance map of the complex illustrates the interconnections between pairs of residues within the system. Covariance analysis is employed to demonstrate the interrelated (red), independent (white), or inversely linked (blue) atomic motions inside the dynamic regions of the intricate molecule (Fig 7F). Ichiye and Karplus calculated the correlation matrix by applying Equation 2 to the Cartesian coordinates of Ca [32]. The elastic network model characterizes the complex’s atoms’ interactions. Each point on the graph represents a spring that connects the corresponding pair of atoms. The color of the dots indicates their level of stiffness. In Fig 7G, lighter shades of grey indicate more flexible areas, while darker shades suggest stiffer regions.

### 3.12. Advanced MD trajectory analysis

**Fig 8:**
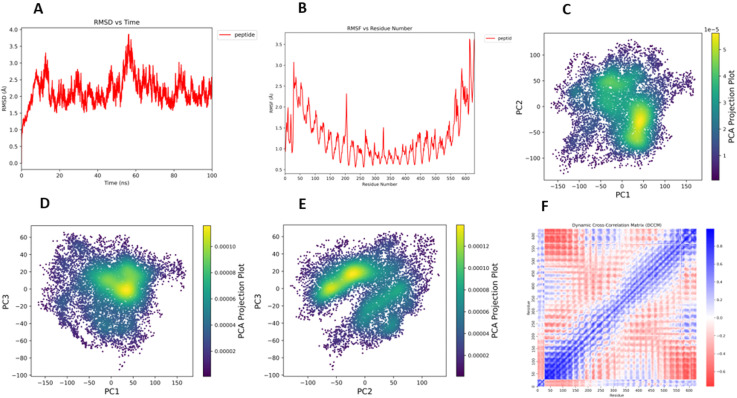
Advanced molecular dynamics trajectory analysis of the ST-MEVC–TLR4 complex. **(A)** Root Mean Square Deviation (RMSD) plot over 100 ns shows the system reaching equilibrium, with fluctuations stabilizing between 1.5–3.0 Å, indicating overall complex stability. **(B)** Root Mean Square Fluctuation (RMSF) analysis highlights residue-level flexibility, with pronounced mobility at the N- and C-termini and loop regions, while binding interface residues remain relatively rigid. **(C–E)** Principal Component Analysis (PCA) projections along PC1 vs. PC2 **(C)**, PC1 vs. PC3 **(D)**, and PC2 vs. PC3 (E) reveal dominant collective motions and conformational clustering, indicating restricted exploration of conformational space and a stable dynamic landscape. **(F)** Dynamic Cross-Correlation Matrix (DCCM) analysis illustrating positively correlated motions (blue) and anti-correlated motions (red) between residue pairs, supporting cooperative dynamics that underpin complex stability.

The 100 ns MD simulation revealed the stability and dynamic behavior of the ST-MEVC–TLR4 complex. The RMSD trajectory (Fig 8A) stabilized between **1.5–3.0 Å**, indicating that the system reached equilibrium after the initial equilibration phase. Minor fluctuations observed around 55–65 ns rapidly stabilized, confirming the robustness of the interaction.

The RMSF profile (Fig 8B) showed that most residues fluctuated within **0.8–2.0 Å**, with peaks at the N- and C-terminal and loop regions, as expected for solvent-exposed segments. Importantly, residues at the vaccine–TLR4 binding interface exhibited minimal fluctuations, reinforcing the stability of the receptor–ligand interaction.

The DCCM analysis (Fig 8F) demonstrated correlated motions (blue) between residues at the binding interface and anti-correlated motions (red) at distal regions, suggesting cooperative conformational dynamics that stabilize the complex.

Finally, PCA analysis (Figs 8C,8D,8E) revealed that the trajectory was dominated by a few principal components, with conformational clustering around stable basins. This indicates that the ST-MEVC–TLR4 complex explores limited conformational space during the 100 ns trajectory, further validating its dynamic stability.

## 4. Discussion

Modern genome sequencing techniques have revolutionized the fight against infectious diseases, enabling the development of novel approaches. Developing vaccines that target specific pathogens is one of the most effective strategies in preventing diseases. Traditional vaccine development methods remain widely applied and effective against numerous diseases. However, advancements in sequencing have paved the way for more targeted and innovative solutions. Similar advances in antigen and antibody-based strategies have also accelerated vaccine and diagnostic development for emerging pathogens such as monkeypox, demonstrating how modern immunotechnology can rapidly support global outbreak response [54].

Applications of in silico procedures can help prevent issues arising from traditional vaccine creation methods. Additionally, these methods are less expensive, more efficient, and yield more precise outcomes [48]. Due to its simplicity, great specificity, and minimal risk, the computational method of vaccine creation is attracting significant attention in the scientific community. Currently, there is no vaccination against *S. typhimurium.* A safe and effective vaccination is crucial for the optimal management of periodontitis. Compared to conventional immunizations, subunit multi-epitope vaccines provide several advantages. In silico vaccine development helps avoid unwanted side effects, such as autoimmunity and immune evasion, by training the host immune system to focus on the most protective antigenic epitopes [49,52]. Similar microbial interactions have been shown in other infections, such as in *C. difficile* infection in Crohn’s disease, where intestinal colonization by Veillonella promotes infection [53], highlighting the need to consider microbial dynamics when designing vaccines.

Furthermore, computational vaccines are safer and more stable than conventional vaccines. However, while vaccines are generally safe, rare immune-mediated complications, such as hemophagocytic lymphohistiocytosis, Sweet syndrome, and Vogt Koyanagi Harada disease, have been reported following SARS-CoV-2 vaccination. Although these events remain extremely uncommon but underscore the importance of rational and precision-based vaccine design approaches to minimize adverse immune responses [55–57].

Epitope predictions have guided the development of several vaccines that provide immunity against human illnesses. One of these initiatives is developing an epitope-based potential vaccine candidate against *Plasmodium vivax.* 2019 saw the testing of a multi-epitope candidate vaccination that was predicted computationally against Acinetobacter baumannii [50,58,59], and in mice, it triggered a robust immune response specific to IgG antibodies. Computationally generated B-cell epitopes have also been assessed for *Trypanosome vivax* diagnostics [51,60–62]. These tactics have been used against several human pathogenic viruses, such as the Ebola virus [52], the Marburg virus [59], the Crimean–Congo hemorrhagic fever virus [58], and the Mokola Rabies virus [60,63],

In our study, we employed a subtractive proteomics strategy to identify *S. typhimurium* antigenic proteins. The subtractive proteomics pipeline found four proteins to fulfill the predetermined requirements for an appropriate protein. Phage integrase protein (Q8ZNX5), proton dehydratase medium subunit (O31041), cytochrome BD2 subunit I (Q8ZRG3), and tryptophan synthase beta chain (P0A2K1) are the UniProt IDs for these proteins. The proteins identified as promising candidates for vaccine development through the subtractive proteomics pipeline are non-homologous to humans, poisonous, and antigenic. These proteins are crucial for the pathogen’s survival. After selection, a protective vaccination was developed by applying immunoinformatics approaches to these four proteins.

Predicting MHC class I and class II binding epitopes was the first step in the immunoinformatics pipeline. The immune system’s T-cells have a remarkable capacity to detect MHC class I and MHC class II epitopes. T-cell receptors (TCRs) recognize antigenic peptides presented by MHC molecules on the surface of host cells. MHC class I molecules, present on all nucleated cells, display endogenous peptides to cytotoxic T lymphocytes (CTLs). Conversely, MHC class II molecules present exogenous peptides to helper T-cells. Our vaccination design includes various B and T-cell epitopes for immune activation [64]. The calculated molecular weight of our vaccine is 41.45 kDa, which falls within the 30–60 kDa range that is ideal for a vaccine protein. Its theoretical PI of 9.71 indicates that it is quite simple. The aliphatic index score, connected to the vaccine’s thermal stability, reflects the amount of aliphatic side chains in the vaccine. Vaccines are considered safe for human consumption when their Instability Index scores are below 40 and stable at or above 31.37. To produce effective vaccines, it is essential to comprehend the structural interactions between receptor molecules and antigens [65]. The vaccine’s 3D structures were predicted and fine-tuned through enhanced structural analysis. The Ramachandran plot’s favorable region revealed the greatest number of residues. The high-quality expected structures of the multi-epitope vaccine constructs were verified using ERRAT and ProSAweb predictions. The vaccine’s interaction with human TLR4 was examined using the Cluspro service.

Molecular dynamics (MD) simulations provided deeper insight into the stability of the vaccine–receptor interaction. The RMSD profile demonstrated that the complex remained stable between 1.5–3.0 Å over 100 ns, while RMSF analysis revealed that flexibility was restricted mainly to terminal and loop regions. Critically, residues at the binding interface showed minimal fluctuation, underscoring stable epitope–receptor contacts. Dynamic Cross-Correlation Matrix (DCCM) analysis confirmed correlated motions in the binding interface, while Principal Component Analysis (PCA) indicated that the complex explored a limited conformational space, clustering around stable states. These findings strengthen the docking analysis and validate that the ST-MEVC–TLR4 complex is dynamically robust and capable of maintaining productive interactions with immune receptors. The vaccine was found to be substantially expressed, as evidenced by the correlation between protein expression and the CAI value and the GC contents of the reverse-translated optimized sequence; these correlations were obtained by optimizing codons using the Jcat server.

Vaccines typically undergo extensive testing in multiple animal models before human use. However, differences between species can lead to reduced performance in humans despite good responses in animals. Consequently, a safe, stable, and very immunogenic vaccine against *S. typhimurium* infections was developed in this study using dependable immunoinformatics and subtractive proteomics approaches. Conventional wet-lab vaccine development is a lengthy and resource-intensive process, often requiring 10–15 years to progress from antigen discovery to licensed vaccines, with costs frequently exceeding several hundred million USD due to extensive experimental phases, pre-clinical models, and multi-stage clinical trials [66]. In contrast, the presented in silico immunoinformatics pipeline enables the rapid identification and prioritization of antigenic targets within a few months at a fraction of the cost, relying on computational tools to screen, model, and validate candidates before wet-lab experimentation. While experimental studies remain indispensable for confirming efficacy and safety, such computational approaches significantly reduce the time and financial burden associated with the discovery phase and streamline the subsequent transition to laboratory validation. Such validation is essential because computational predictions, while powerful, may generate false positives or overlook conformational effects seen only in wet-lab settings. Future work should confirm these findings using in vitro and in vivo studies. In vitro validation was possible for dendritic cell and peripheral blood mononuclear cell (PBMC) assays (antigen presentation, cytokine release and T-cell activation). The vaccine candidate may also be tested in relevant cell lines, THP-1 monocytes and Jurkat T-cells for functional immunological responses. In vivo testing often involves murine models (e.g., BALB/c or C57BL/6) to measure immunogenicity, antibody titres and T-cell responses. Advanced immunoassays as ELISA, ELISPOT and flow cytometry could be employed for measurement of immune response and toxicity/safety assessments should not be neglected at the same time. These studies in future will offer experimental evidence required to convert the predictions from computational approach into a functional vaccine candidate.

We acknowledge that more recent ML-based predictors such as DeepVacPred, iVAX, and transformer-based models are emerging in the field and may provide additional predictive power. However, for this study we selected widely used and extensively benchmarked tools (VaxiJen, AlgPred2, ToxinPred, ABCPred) to ensure reproducibility, comparability with prior immunoinformatics research, and methodological robustness. Future work may integrate deep learning–based predictors as these platforms mature and undergo broader validation.

## 5. Conclusion

The current study reports reverse vaccinology and immunoinformatic approaches to identify potential therapeutic vaccine targets using proteomic data of *S. typhimurium*. A multi-epitope vaccine was developed by selecting overlapping T-cells and B-cell epitopes from the key proteins. The immunogenicity was enhanced by linking them with appropriate adjuvants and linker peptide sequences. The immunoinformatic analysis confirmed that the designed vaccine is optimal and capable of efficient expression in *E. coli*. Furthermore, immunological simulations, (MD) simulations, and molecular docking studies demonstrated that the designed vaccine has a strong binding affinity to human immune cell receptors. The current study concludes that vaccines designed through reverse vaccinology and immunoinformatic approaches strongly trigger cell-mediated immune responses in humans against *S. typhimurium*. Further in vitro and in vivo studies are recommended to validate its efficacy.

## Supporting information

S1 Fig Structure of all predicted CTL epitopes mapped on the modeled proteins (green). (TIF)

S2 Fig Structural representation of all the predicted HTL epitopes onto the modeled proteins (yellow). (TIF)

S3 Fig Visualization of all potential B-Cell epitopes on the modelled proteins (red). (TIF)
