# Supplementary figures and images for: Immunoinformatics-driven design of multi-epitope vaccine targeting antibiotic-resistant Salmonella typhimurium
Source: PLoS One. 2026 Feb 26;21(2):e0342426. doi: 10.1371/journal.pone.0342426 (PMC12944783; doi:10.1371/journal.pone.0342426)

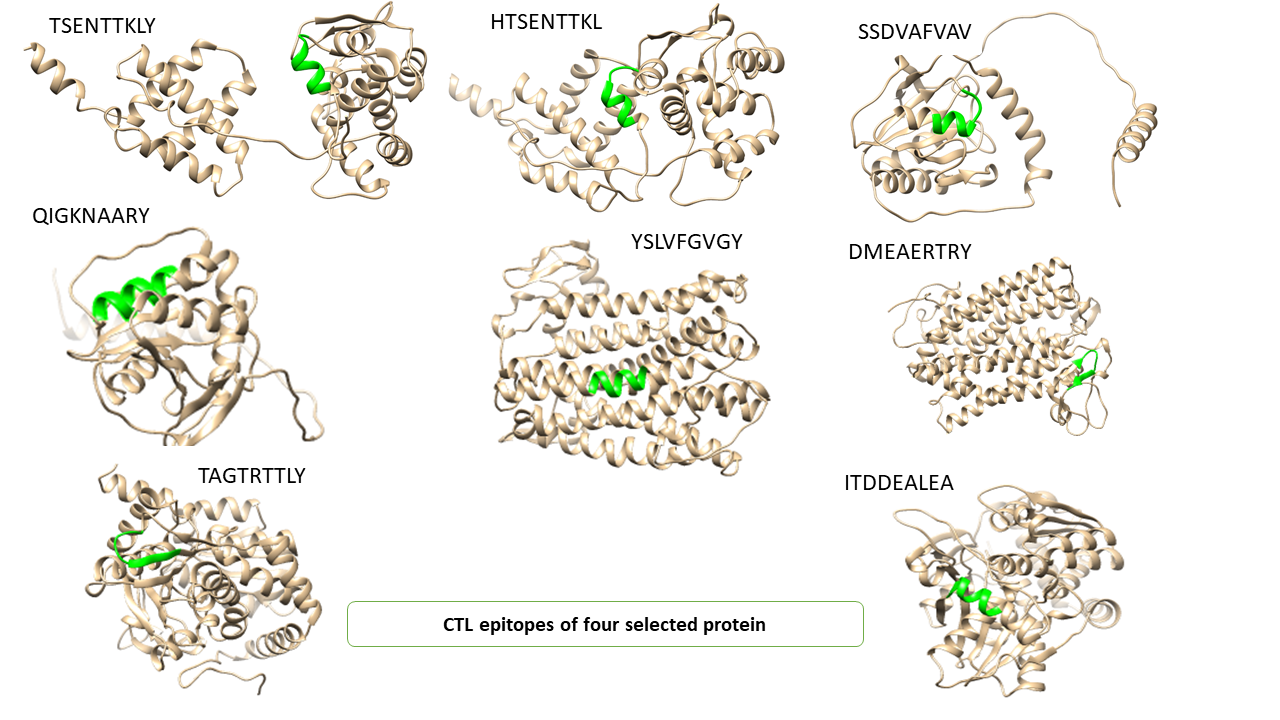

Supplement: S1 Fig — (TIF) [file pone.0342426.s001.tif]

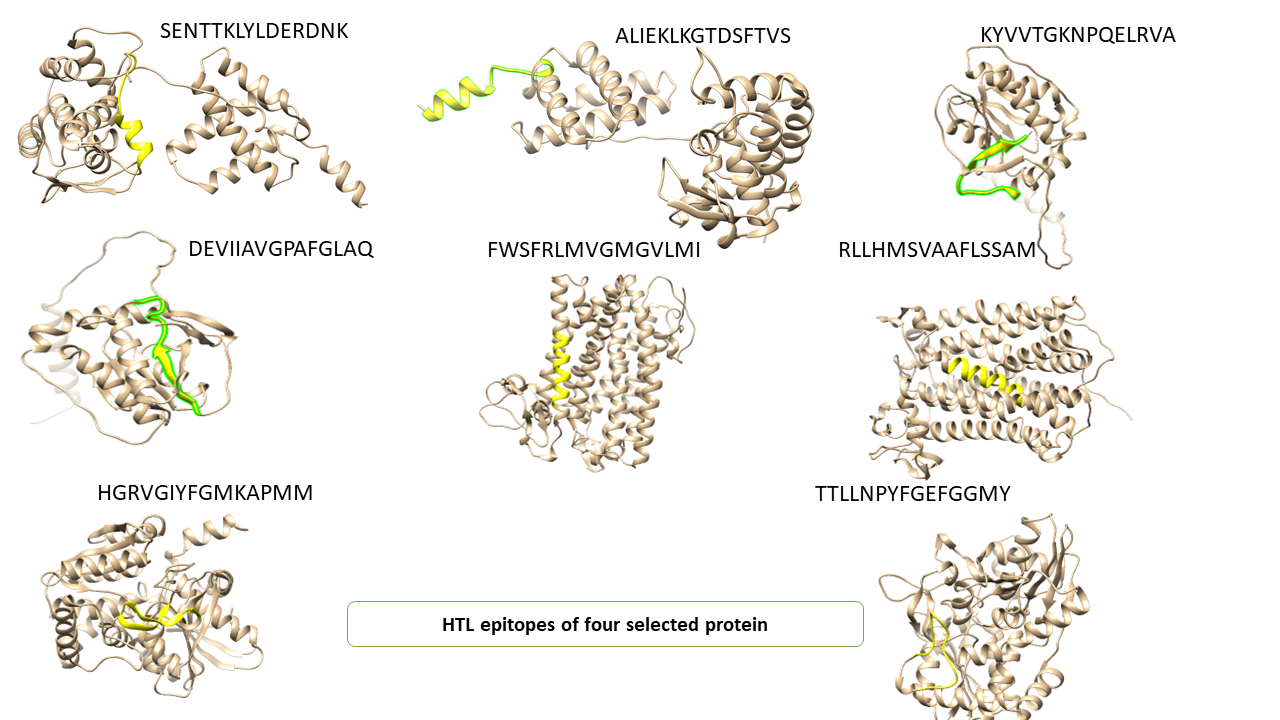

Supplement: S2 Fig — (TIF) [file pone.0342426.s002.tif]

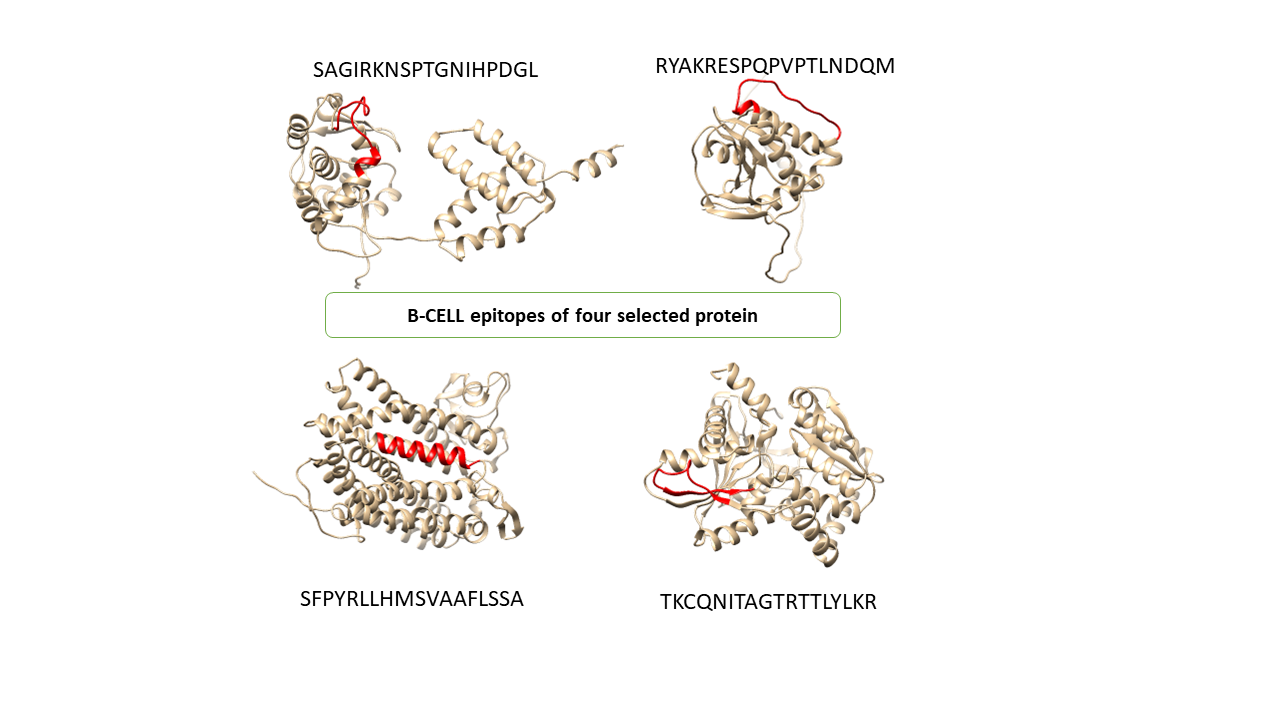

Supplement: S3 Fig — (TIF) [file pone.0342426.s003.tif]
